# Supplementary material for: Gaze Fixation and Visual Searching Behaviors during an Immersive Virtual Reality Social Skills Training Experience for Children and Youth with Autism Spectrum Disorder: A Pilot Study
Source: Brain Sci. 2022 Nov 18;12(11):1568. doi: 10.3390/brainsci12111568 (PMC9688560; doi:10.3390/brainsci12111568)
Supplement: Supplementary file 1 [file brainsci-12-01568-s001.zip › brainsci-1969461-supplementary.pdf]

Supplementary Table S1: The number of gaze on object and avatar (non-face area), mean (SD).

Gaze Area	All participants (N = 10)	Mild (n = 6)	Moderate (n = 2)	Severe (n = 1)
Object				
Blackboard	15.0 (16.5)	5.3 (3.2)	16.0 (-)	- (-)
Chair 1	16.2 (18.4)	15.8 (23.7)	15.0 (-)	- (-)
Chair 2	13.5 (17.3)	9.3 (12.7)	8.0 (-)	1.0 (-)
Controller right	19.0 (-)	19.0 (-)	- (-)	- (-)
Desk 1	27.2 (18.1)	27.0	14.0	- (-)
Desk 2	4.2 (2.8)	5.5	1.0	2.0 (-)
Globe	27.8 (28.3)	13.3	22.0	- (-)
Light switch	2.0 (-)	2.0 (-)	- (-)	- (-)
Poster	47.5 (45.4)	52.5 (57.6)	41.0 (-)	- (-)
Teacher chair	11.3 (10.1)	3.0 (2.8)	16.0 (-)	- (-)
Teacher desk	36.2 (33.7)	19.7 (15.0)	29.0 (-)	- (-)
Wall collider	32.6 (37.1)	15.5 (16.3)	32.0 (-)	5.0 (-)
Wall collider 1	78.8 (56.9)	89.0 (52.2)	57.0 (-)	4.0 (-)
Wall collider 2	142.8 (141.7)	229.8 (149.5)	39.0 (53.7)	6.0 (-)
Wall collider 3	23.5 (16.5)	29.0 (-)	18.0 (-)	4.0 (-)
Floor collider	320.3 (205.9)	260.3 (139.2)	437.5 (410.8)	205.0 (-)
null	5930.8 (9524.7)	9379.3 (11275.3)	304.0 (63.6)	2108.0 (-)
Avatar				
Left hand area	25.0 (21.2)	10.0 (12.8)	49.0 (0.0)	- (-)
Right hand area	29.5 (24.5)	22.8 (13.3)	74.0 (-)	- (-)

Supplementary Table S2: The duration of gaze on object and avatar (non-face area), mean (SD), seconds.

Gaze Area	All participants (N = 10)	Mild (n = 6)	Moderate (n = 2)	Severe (n = 1)
Object				
Blackboard	4.9 (8.2)	7.2 (1.2)	4.4 (8.7)	- (-)
Chair 1	8.0 (21.7)	5.8 (9.7)	23.9 (49.7)	- (-)
Chair 2	4.5 (11.1)	7.6 (1.8)	3.9 (4.5)	15.6 (0.0)
Controller right	2.1 (1.9)	2.1 (1.9)	- (-)	- (-)
Desk 1	9.5 (18.8)	11.3 (2.2)	16.0 (19.4)	- (-)
Desk 2	1.6 (1.3)	1.9 (1.7)	1.1 (-)	1.1 (0.0)
Globe	14.8 (28.4)	21.9 (4.0)	25.8 (37.1)	- (-)
Light switch	3.9 (3.9)	3.9 (3.9)	- (-)	- (-)
Poster	5.7 (9.5)	5.4 (8.7)	9.6 (14.8)	- (-)
Teacher chair	3.8 (5.7)	3.2 (2.9)	5.3 (8.4)	- (-)
Teacher desk	13.0 (25.8)	14.4 (31.0)	25.2 (37.7)	- (-)
Wall collider	31.0 (57.3)	53..3 (7.9)	34.4 (68.0)	2.4 (3.0)
Wall collider 1	12.6 (22.5)	12.8 (2.0)	20.9 (27.9)	1.1 (0.0)
Wall collider 2	18.2 (56.6)	18.0 (19.9)	32.9 (60.9)	1.3 (0.4)
Wall collider 3	11.0 (14.7)	12.2 (58.6)	16.7 (14.9)	1.1 (0.0)
Floor collider	32.8 (72.4)	24.6 (58.7)	51.8 (93.3)	13.8 (57.8)
null	1.1 (2.1)	1.1 (1.8)	1.5 (9.5)	1.2 (3.9)
Avatar				

Left hand area	2.2 (3.0)	3.0 (5.3)	2.1 (1.9)	- (-)
Right hand area	2.9 (3.8)	3.2 (4.1)	2.6 (3.6)	- (-)
